# Dynamics of positional information in the vertebrate neural tube

**DOI:** 10.1098/rsif.2024.0414

**Published:** 2024-12-11

**Authors:** Anđela Marković, James Briscoe, Karen M. Page

**Affiliations:** ^1^ Department of Mathematics, University College London, London WC1E 6BT, UK; ^2^ The Francis Crick Institute, London NW1 1AT, UK; ^3^ Institute of Physics of Living Systems, University College London, London WC1E 6BT, UK

**Keywords:** positional information, neural tube, information theory, tissue development, morphogen

## Abstract

In developing embryos, cells acquire distinct identities depending on their position in a tissue. Secreted signalling molecules, known as morphogens, act as long-range cues to provide the spatial information that controls these cell fate decisions. In several tissues, both the level and the duration of morphogen signalling appear to be important for determining cell fates. This is the case in the forming vertebrate nervous system where antiparallel morphogen gradients pattern the dorsal–ventral axis by partitioning the tissue into sharply delineated domains of molecularly distinct neural progenitors. How information in the gradients is decoded to generate precisely positioned boundaries of gene expression remains an open question. Here, we adopt tools from information theory to quantify the positional information in the neural tube and investigate how temporal changes in signalling could influence positional precision. The results reveal that the use of signalling dynamics, as well as the signalling level, substantially increases the precision possible for the estimation of position from morphogen gradients. This analysis links the dynamics of opposing morphogen gradients with precise pattern formation and provides an explanation for why time is used to impart positional information.

## 1. Introduction

The process of tissue development is remarkably reliable and precise. It involves the generation of distinct cell types in a characteristic and reproducible arrangement to form organized patterns of cell fates. In an influential essay that describes this process, Lewis Wolpert introduced the concept of ‘positional information’ [1]. This proposed that positional information is derived from signals, usually referred to as morphogens, which form concentration gradients in the tissue, with cells measuring the local concentration and interpreting it to select the appropriate cell fate for that position. In this view, concentration thresholds define boundaries that distinguish adjacent sets of cell types.

**Figure 1.**
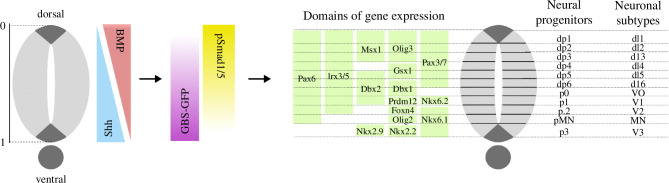
The morphogen Shh, emanating from the ventral pole, and the morphogen BMP, secreted from the dorsal pole of the neural tube, form antiparallel gradients along the dorsal–ventral axis. These extracellular gradients are translated into intracellular gradients of the activity of Gli and Smad transcription factors, regulated by Shh and BMP signalling, respectively. Their activity is measured using GBS-GFP, a Gli reporter, and pSmad1/5, the activated version of Smad1/5. The Gli and Smad transcription factors control gene regulatory networks that specify the domains of gene expression along the axis, and hence partition the neural tube into 11 discrete domains of molecularly distinct neural progenitors. In this way, the positions at which distinct neuronal subtypes will be generated are determined.

The dorsal–ventral patterning of the vertebrate nervous system is a well-established system for studying morphogen-driven patterning [2]. Signals emanating from two opposing sources—the dorsal roof plate secreting the morphogen BMP, and the ventral floor plate secreting the morphogen Shh—form antiparallel gradients that partition the neural tube into 11 discrete domains of molecularly distinct neural progenitors arrayed along the dorsal–ventral axis (figure 1). Neural progenitors respond to morphogen signalling by controlling a gene regulatory network that specifies the pattern of gene expression along the dorsoventral axis and hence the positions at which distinct neuronal subtypes are generated. Gene expression and consequently cell fate appear to be specified by a combination of Shh and BMP signalling [3]. Positions of several gene expression boundaries have been experimentally measured [3–6] and this indicates that the boundaries are specified with a high degree of precision, resulting in sharply defined transitions in gene expression and only moderate intermixing of cell types at domain boundaries.

To explore the amount of positional information that can be obtained from the two morphogens, we took advantage of tools from *information theory* [7]. These have been used previously to study information transmission through signalling pathways and gene regulatory networks [8–16], including positional information transmission by the morphogen Bicoid, which governs the anterior–posterior patterning of the *Drosophila* blastoderm [17–21]. The variability between embryos of the Bicoid gradient and the expression levels of the genes it controls were used to connect positional information to experimental data at a specific developmental time point. This revealed that Bicoid has a lower precision than its target genes [17], leading to the possibility that other sources of information or mechanisms are involved, such as continuous, rather than instantaneous assessment of Bicoid concentration [22].

The *Drosophila* blastoderm differs in several ways from many developing tissues. It is a syncytium. This allows Bicoid to regulate gene expression directly without an intervening signal transduction pathway. Most developing tissues, including the neural tube, are cellularized and morphogens spread extracellularly to activate intracellular signalling pathways that control gene expression [23,24]. In addition, the patterning of the blastoderm occurs in the absence of tissue growth [25]. Most tissues grow as they are patterned, and morphogen gradients do not always scale with growth [6,23]. This, along with other factors such as changes in the production of the signal, results in the gradient changing as the tissue is patterned [23]. Consequently, there is no straightforward correlation between the concentration of morphogens, their location in tissue and cell fate allocation. Indeed, the duration of morphogen signalling has also been shown to influence cell fate decisions [4,26–28]. It is unclear, given the dynamics and heterogeneity and fluctuations in the spread and interpretation of a morphogen, how a graded morphogen produces the precise patterns of cell fate decisions that are observed in developing tissues.

Despite the prominence of Wolpert’s positional information concept, it has become evident that relying solely on morphogen concentration does not provide a comprehensive explanation for understanding morphogen gradients and their role in tissue development [24]. The ‘progressive and dynamic manner’ [26] in which the domains of neural progenitors appear suggests new ways of encoding positional information. Here, we set out to extend the information-theoretic approach by adapting methods that accommodate dynamics to analyse positional information in the neural tube. We wanted to explore the impact of signalling dynamics on positional precision and introduce a temporal dimension to the concept of positional information.

## 2. Results

### 2.1. Quantifying positional information with information theory

We first set out to quantify how much positional information the Shh and BMP morphogens provide in the neural tube. The signalling pathways of these morphogens represent complex, noisy channels through which the cells receive information about their positions [23]. Information theory describes the input and the output of any noisy channel as two random variables, X and Y, respectively [29]. The noise in the channel leads to a distribution of possible output responses produced by the same input message [16], and the uncertainty in the input arises from the noise in its source as well as other features of the system. Let X be a discrete random variable that can take values $x_{1},x_{2},\ldots ,x_{m}$, with the probability distribution $P_{X}(X)=(P_{X}(x_{1}),\ldots ,P_{X}(x_{m}))$, and let Y be a continuous random variable, with the probability distribution $P_{Y}(Y)$ and Y as a space of all possible values of Y. The main measure of information that we use here, the *mutual information* (MI) between two random variables X and Y, MI(X,Y), is defined by [30]


(2.1) $$\begin{array}{c} MI(X,Y)=\sum_{i=1}^{m}\int_{Y}P(x_{i},y)\log_{2}\frac{P(x_{i},y)}{P_{X}(x_{i})P_{Y}(y)}\;dy \end{array},$$

where


$$P(x_{i},y)=P(y|X=x_{i})P_{X}(x_{i})=P(x_{i}|Y=y)P_{Y}(y).$$

MI(X,Y) measures to what extent X and Y depend on each other, being 0 when they are independent, and captures any kind of correlation between the input and the output [19]. It indicates how much the uncertainty about the value of the random variable X is reduced by knowing the state of Y, that is, how much information we obtain about the input after looking at the output [29]. More precisely, MI(X,Y) can be interpreted as the average number of binary (yes/no) questions about the value of X that are answered after observing Y. Each question divides possible values of X into two equally probable, mutually exclusive classes based on the values of Y associated with them. Hence, $2^{MI(X,Y)}$ gives us the average number of equiprobable classes of the values of the input X that produce classes of the values of the output Y that can be distinguished without error [31]. Two individual values of X, $x_{i}$ and $x_{j}$, are said to be resolved if their corresponding output distributions $P(Y|X=x_{i})$ and $P(Y|X=x_{j})$ do not overlap [32]. When all the input values have the same probability, $P_{X}(x_{1})=\cdots =P_{X}(x_{m})$, then the MI depends solely on the overlap between their output distributions and provides a measure of the noise that leads to these overlaps and prevents the system from perfectly resolving each input value.

**Figure 2.**
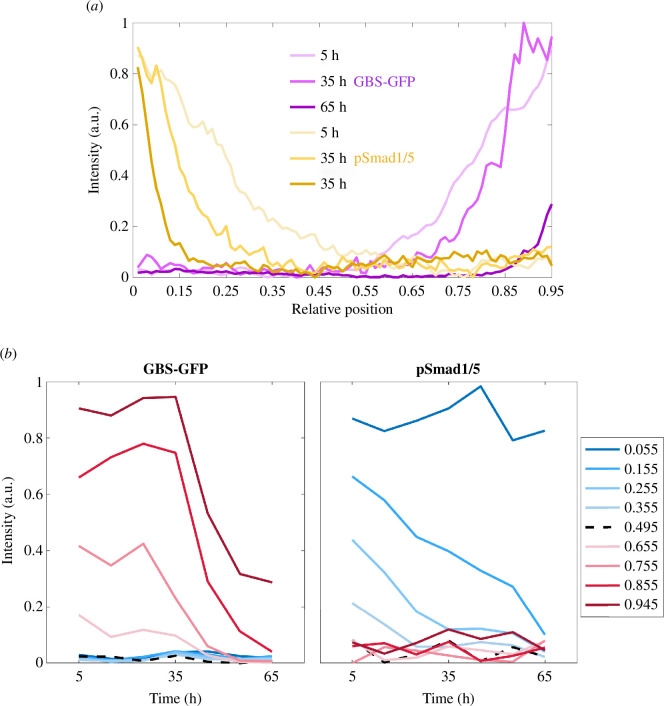
(*a*) The mean gradients of the activity of GBS-GFP, the reporter of Shh signalling, and the gradients of the activity of pSmad1/5, the transcriptional effectors of the morphogen BMP, along the relative dorsal–ventral length of the neural tube at 5, 35 and 65 h during development. (*b*) The mean levels of GBS-GFP and pSmad1/5 over time at the indicated relative positions.

The outputs of the Shh and BMP signalling pathways are the activities of their intracellular transcriptional effectors. These were measured in the mouse neural tube by Zagorski *et al*. [3]. Shh signalling results in the intracellular activation of Gli transcription factors, the activity of these being measured using a transcriptional reporter, GBS-GFP. BMP signalling activates Smad transcription factors, which were measured using an antibody specific for phosphorylated SMAD1/5, pSmad1/5, the activated version of the BMP transcriptional effectors. Hence, we take the activities of GBS-GFP and pSmad1/5 to be the outputs of the Shh and BMP signalling pathways, respectively (figure 1). To quantify the positional information provided by the signalling of Shh and BMP morphogens in the neural tube, we computed MI(X,Y) where the input X represents different relative positions $x_{1},\ldots ,x_{m}$ along the dorsal–ventral length of the neural tube, and the output Y the levels of GBS-GFP and pSmad1/5 activity measured at these positions (figure 2). Following the ideas in [17,19], we argue that this definition of MI(X,Y) is a proper measure of positional information in the neural tube.

More precisely, the input probability distribution $P_{X}(X)$ represents the positions of cells along the dorsal–ventral axis. Since cell density is approximately constant along this axis, we take the distribution $P_{X}(X)$ to be uniform [6]. The relative dorsal–ventral length of the neural tube from position 0.055 to 0.945, where position 0 corresponds to the dorsal end and 1 to the ventral end, is divided into bins of width 0.05 with a distance of 0.01 between them. This resulted in 15 bins and therefore 15 input values denoted sequentially as $x_{1},\ldots ,x_{15}$ (electronic supplementary material, figure S1). Using the measurements of the neural tube length over time from [3], the average cell diameter, 4.9 μm [6], is 0.05 of the relative length at 5 h of neural tube development, the first time point at which the signals are measured. The average absolute length of each bin grows over time, but during the first 30 h of development, this length is less than 2 average cell diameters, and at 35 h it is approximately 2 cells.

Zagorski *et al*. [3] measured the levels of GBS-GFP and pSmad1/5 every 0.01 of relative length from position 0.055 to 0.945, along the dorsal–ventral length of the neural tube in a single embryo. Hence, one of our 15 bins, $x_{i}$, comprises 6 single relative positions at which the signals are measured and the output values corresponding to $x_{i}$ are concatenated measurements of the signals made at those 6 positions in all embryos. As in Zagorski *et al*. [3] and Dubuis *et al*. [17], we focus on how much information there is at fixed relative positions in the tissue. The 15 bins allow us to determine this (see electronic supplementary material for details) and are independent of the progenitor domains, which are not of equal proportions and change during development [6].

Information-theoretic tools have been used before to study dynamic signals transmitting information to cells about their environment [33–35]. This has led to the idea that a single cell monitors signals over time to overcome noise [33] and that information can be encoded in different dynamical patterns of signals [36]. Therefore, the output values were represented as vectors of cellular responses at different time points. To explore the impact of morphogen signalling dynamics on positional precision, we took a similar approach and used the levels of GBS-GFP and pSmad1/5 measured at a particular relative position at successive time points, $t_{1},t_{2},\ldots$, during neural tube development. Hence, a single output response to an input $x_{i}$ becomes a vector of the form $y^{i}=\left(y_{G}^{i}(t_{1}),y_{G}^{i}(t_{2}),\ldots ,y_{S}^{i}(t_{1}),y_{S}^{i}(t_{2}),\ldots \right)$, where G denotes GBS-GFP and S pSmad1/5. Note that the signals are only measured once in an embryo and then the temporal trajectory is inferred from measurements at equivalent positions in embryos of sequential time classes (see electronic supplementary material and [3,6] for details).

To compute MI, we took advantage of a recently developed framework proposed by Jetka *et al*. [32]—SLEMI (statistical learning estimation of mutual information)—which simplifies the calculation of MI for high-dimensional output. The method involves a classifier that uses a logistic regression model to estimate $P(x_{i}|Y=y)$, the probability of being at a position $x_{i}$ after observing a signal level y, from the given data such that the probability of being at the true position for a given signal is maximized. Therefore, SLEMI is similar to ‘decoding-based information estimators’, described in [37], which use machine learning classifiers. We compared SLEMI with one of them, the decoding-based method proposed by Granados *et al*. [34]. Jetka *et al*. [32] demonstrated the validity of their method and showed its advantages over the most commonly used method for computing MI, k-nearest neighbour (kNN). We also applied the kNN method to our data. The three computational tools give similar results (electronic supplementary material, figure S2) and a more detailed comparison can be found in the electronic supplementary material. Overall, the comparison of the tools showed that SLEMI is the most appropriate one for our dataset given the chosen number of input values and the dimensionality of output responses.

The calculation of MI led to the values of $2^{MI(X,Y)}$ which, in this case, represent the average number of spatial regions along the dorsal–ventral axis of the neural tube that can be clearly delineated based on received signal levels. Since the positions are uniformly distributed, these regions are of equal length.

### 2.2. Information from morphogen gradients at single time points

We computed MI between the positions along the dorsal–ventral axis of the neural tube and the levels of GBS-GFP and pSmad1/5 activity measured at these positions. We considered the levels of each signal separately and the combined levels. The data consist of 102 measurements of each of the two signals measured in each of the 15 spatial bins at each of the 7 time points 5, 15, 25, 35, 45, 55 and 65 h during neural tube development. We first calculated the MI for each time point by using only the signal levels measured at the corresponding time point. We then considered series of signal levels at successive time points and computed the time-series MI from the first time point up to each later time point. For example, the values measured at 5, 15, 25 and 35 h were used to compute the MI for the 35 h time course.

**Figure 3.**
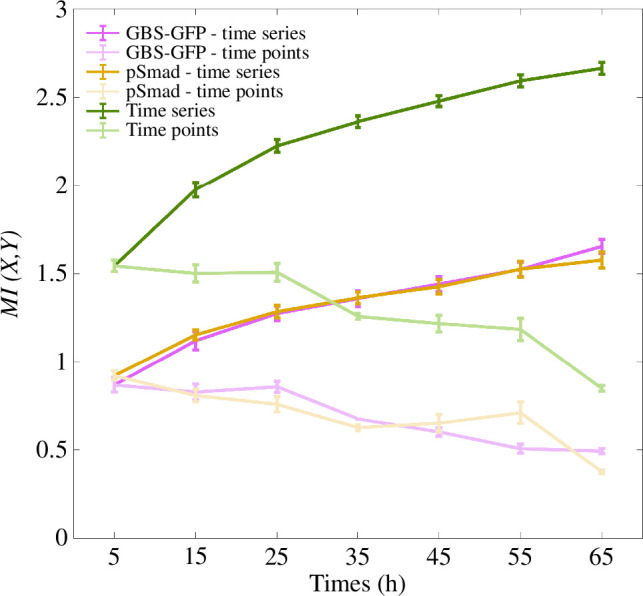
Mutual information (MI) between the 15 positions along the dorsal–ventral axis and the levels of the signals GBS-GFP and pSmad1/5 measured at these positions. The values of MI are first computed using the levels of GBS-GFP and pSmad1/5 separately, and then based on the levels of the two signals combined (where it is not otherwise denoted). The levels were measured at seven time points during development. The time-point MI was computed using signal levels measured at each time point individually. The time-series MI is computed using the series of signal levels from the first time point up to each later point. The values are averaged over different embryos at a single time point and over different combinations of embryos when time series is considered. The error bars are the standard deviations. Units are bits.

The values of time-point MI are highest at the first three time points, 5, 15 and 25 h (figure 3). However, when taking GBS-GFP and pSmad1/5 separately, the time-point MI does not reach 1 bit, the value needed to distinguish two regions. This indicates that at a single point in time, on average, the information from either Shh or BMP signalling is insufficient to create a sharp boundary in the neural tube. On the other hand, the MI for the two signals combined is higher and shows that, at these first three time points, there is almost enough information to be able to resolve three regions ($2^{1.58}\approx 3$). Thus, by sensing both morphogens at a single time point, there is sufficient information to specify two spatial regions.

### 2.3. Temporal trajectories of morphogen gradients increase positional information

The time-series MI increases with the inclusion of an increasing number of time points, indicating that new information continues to accumulate over time. The information accumulated during the first 65 h from only Shh signalling is sufficient to distinguish three regions along the neural tube. For pSmad1/5 activity, the MI values are similar. By contrast, the values of MI for the combined Shh and BMP signalling indicate that the information accumulated during the first approximately 30 h is sufficient to resolve five regions without error ($2^{2.32}\approx 5$). This rises to 2.67±0.03 bits by 65 h ($2^{2.58}\approx 6$). This confirms that the two morphogens acting together carry significantly more information. Overall, the analysis indicates that the combined signalling of the two morphogens over time changed the result from no two regions resolved at a single time point to almost seven distinguishable regions accumulated over 65 h.

Since positional information is used to classify cells into correct domains of gene expression, it is reasonable to ask what the precision is in terms of number of cells. Using the interpretation of $2^{MI(X,Y)}$, the time-series MI for the combined signalling revealed that the average absolute length of the regions that can be perfectly distinguished, in terms of average cell diameters, remains approximately the same during the first 30 h, around 6 cell diameters. After that, the length increases; it is approximately 7 cells for the 35 h time course and 12 cells for 65 h of signalling. If we used the time-point MI, this length would be 6, 8, 10, 16, 20, 26, 42 at the seven time points, respectively. This means that during the first 30 h of development, the average amount of information that is accumulated from Shh and BMP signalling increases proportionally to the tissue growth, maintaining positional precision. This result is in agreement with the claim that the pattern is established during the first approximately 30 h, which was supported by the analysis of Zagorski *et al*. [3] and the experimental evidence of Kicheva *et al*. [6].

This result is dependent on the choice of the time points and this level of precision is lost when we consider the points every 20, 30 or 60 h (electronic supplementary material, figure S3). As we decrease the time step, the total amount of information for the 65 h time course significantly increases. We expect that for a sufficiently small time step, reducing the step further will not add new information. However, we do not have data to determine the exact time step at which this happens. Here, we assume that the signal levels received over time are stored. This comes with a cost, which increases with the dimensionality of the time series, which is, on the other hand, limited by the memory capacity of the cells [35]. Another disadvantage of longer time series is that, since the time points are closer, there will be more redundant information [35]. In our case, considering all the seven time points leads to a high level of redundant information (electronic supplementary material, figure S4), which raises the question of the efficiency of encoding information in this way.

We expected the time-series information to increase with time, and we noticed that the increase in the information becomes smaller at each next time step (electronic supplementary material, figure S5). The results show that the most information is gained early in development, when the gain is also higher with a smaller time step. This is true for the time points 5, 15 and 25 h, or approximately the first 30 h of development. Later, as the redundancy increases, larger time steps bring higher information gain.

### 2.4. Comparison of positional information from time series and time integrals

The dynamics of neural tube patterning challenges both the concept of positional information and the information-theoretic approach to studying dynamic signals. If the duration during which signal levels are maintained above a particular threshold determines position [26], the question is whether a whole vector of signalling dynamics should represent encoded positional information. It has been suggested that the positional information in the neural tube could be encoded as the time integral of signalling [26,28]. Therefore, we considered cumulative level and duration by summing the entries of vectors for each of the two signals and computed MI(X,Y) where a single output response corresponding to an input $x_{i}$ is now $y^{i}=\left(y_{G}^{i}(t_{1})+y_{G}^{i}(t_{2})+\cdots ,y_{S}^{i}(t_{1})+y_{S}^{i}(t_{2})+\cdots \right)$.

**Figure 4.**
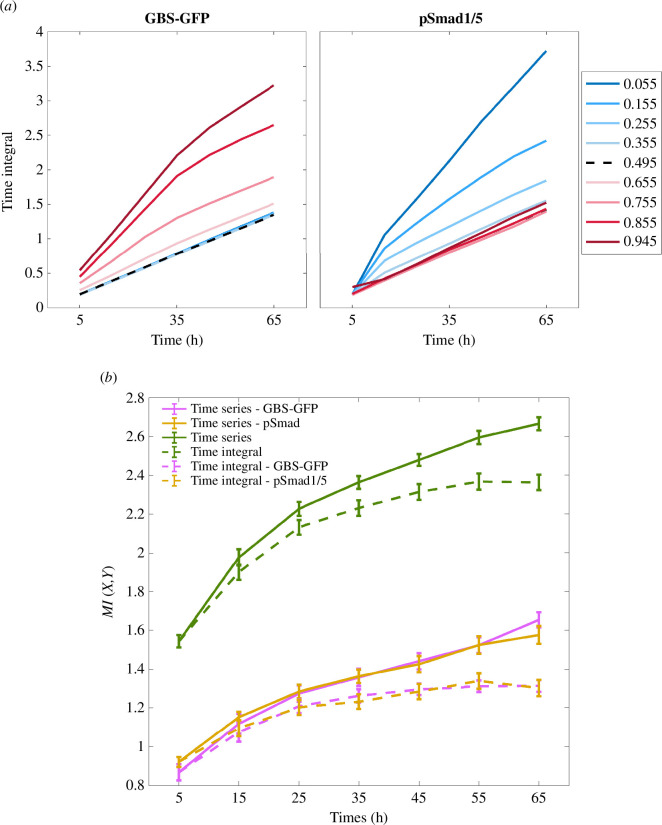
(*a*) The mean values of time integrals of GBS-GFP and pSmad1/5 over time at the indicated relative positions. (*b*) The time-series and the time-integral mutual information using the levels of GBS-GFP and pSmad1/5 separately, and based on the levels of the two signals combined. The time-integral mutual information is measured by summing the signal levels received over time, up to each time point. Units are bits.

This time-integral MI during the first approximately 30 h is slightly lower than the time-series MI (figure 4). The difference between the two slowly increases over time since the time-integral MI plateaus after 35 h, with even a slight decrease at 65 h. If the domains of gene expression are established during the first 30 h, any new information that arrived after that time might not be used. Furthermore, when considering the two signals together, the time-integral MI gives approximately the same level of precision during the first 30 h as the time-series MI, i.e. the absolute length of the regions along the neural tube that can be perfectly distinguished is approximately 6 cells during this time. Later, the length increases and is around 15 cells by 65 h.

Encoding information using combined dynamics of multiple factors has also been considered by Granados *et al*. [34]. They introduced a measure for the degree of redundancy between a pair of transcription factors, which gives 0 when they are completely independent and 0.5 when they are completely redundant. In the time-series scenario, the redundancy between the activities of GBS-GFP and pSmad1/5 is approximately 0.13 during the first 45 h and then increases to 0.18 by 65 h. When considering time integrals of the signals, the redundancy decreases over time to less than 0.1 by 65 h (electronic supplementary material, figure S6).

These results support experimental findings in [26], which suggested that the time integral is a suitable mechanism for encoding positional information in the neural tube.

Although we do not know precisely what features of morphogen signalling cells use to obtain knowledge about their positions, the introduction of time led to considerably more information than if static morphogen gradients were considered. This may explain why developmental systems have been found to respond to duration of morphogen signalling as well as its level. It emphasizes the importance of a definition of positional information that captures this extended morphogen sensing.

### 2.5. Probability distributions of positional assignments

The number of bits does not necessarily indicate the number of regions that are perfectly distinguishable in the neural tube: 1 bit could indicate that two regions are completely resolved or it could indicate that three or more regions are resolved, but with some error and the regions could be of different lengths [38]. As mentioned above, SLEMI tells us how well the true position of an observed signal level can be inferred by estimating the probabilities $P(X=x_{j}|Y=y_{l}^{i})$, for all i,j=1,…,15 and l=1,…,102, where $y_{l}^{i}$ denotes the *l*th output response measured in bin $x_{i}$. Here, a signal level $y_{l}^{i}$ can be either a scalar, when the level of GBS-GFP or pSmad1/5 is taken separately at a single time point or its time integral, or a vector when both signals and/or consecutive time points are considered. Since signal levels are measured at 90 relative positions, every 0.01 of relative length from position 0.055 to 0.945, we find the average probabilities of each of these positions being in each of the 15 bins by averaging $P(X=x_{j}|Y=y_{l}^{i})$ over signal levels measured at that position [21]. We denote these probabilities by $P(X^{*}=x_{j}|x^{i,k})$, where $X^{*}$ is a discrete random variable with values $x_{1},\ldots ,x_{15}$ corresponding to the bins and $x^{i,k}$, for i=1,…,15 and k=1,…,6, denotes one of the six relative positions in a bin $x_{i}$.

**Figure 5.**
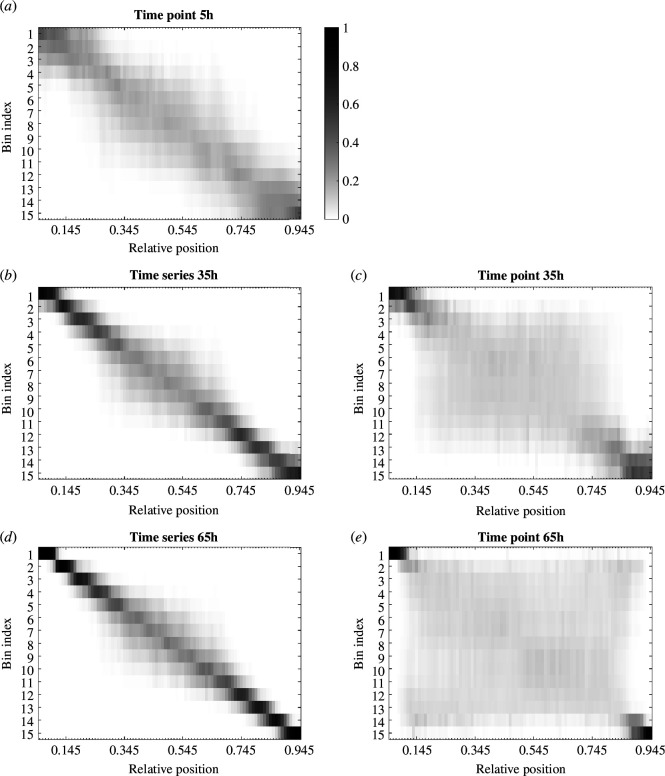
The average probabilities of being in each of the 15 bins after observing levels of the two signals measured at each of the relative positions from 0.055 to 0.945, at time points (*a*) 5, (*c*) 35 and (*e*) 65 h, and for the time series of levels up to (*b*) 35 and (*d*) 65 h.

Using the levels of GBS-GFP and pSmad1/5 activity together at individual time points, the probabilities of classifying relative positions into correct bins along the patterning axis increase at the poles of the neural tube and decrease everywhere else with each next time point (figure 5; electronic supplementary material, figure S7). The lack of scaling of the gradients as the neural tube grows leads to this expansion of the region of low positional information over time (figure 2). On the other hand, the time-series analysis shows that information accumulated during the first 35 h leads to notably higher probabilities of correct positional discrimination along the whole length of the neural tube, particularly for the positions within the dorsal and ventral third. Considering the time integral of signal levels, the information along the neural tube is distributed in the same way as in the time-series scenario. The time integrals of signal levels lead to slightly lower probabilities during the first 35 h, compared to the time-series scenario, and this difference increases over time. More precisely, the highest difference between the time-series and time-integral probabilities after 15, 25 and 35 h is 0.06, 0.09 and 0.09, respectively, while after 65 h it is 0.27 (electronic supplementary material, figure S8).

**Figure 6.**
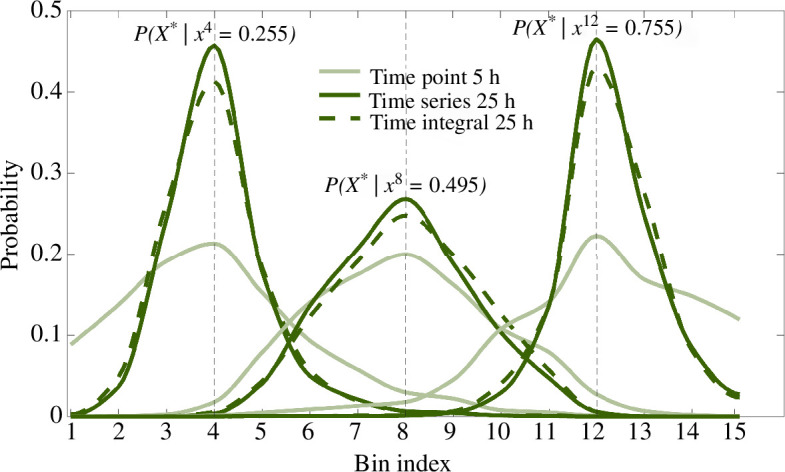
The probability distributions $P(X^{*}|x^{i})$ showing the average probabilities of being in each bin after observing levels of the two signals measured at a relative position $x^{i}$, for $x^{4}=0.255,x^{8}=0.495$ and $x^{12}=0.755$. We computed these at the time point 5 h and for time series and time integral over the first 25 h.

We looked closely at the average distribution $P(X^{*}|x^{i})$, the average probabilities of being in each bin after observing a signal level measured at a single relative position $x^{i}$ within bin $x_{i}$, for i=4, 8, 12 and $x^{4}=0.255,x^{8}=0.495$ and $x^{12}=0.755$, at the time point 5 h and for time series and time integral over the first 25 h (figure 6).

The increase in the probability of being in the correct bin between 5 and 25 h is particularly noticeable for the positions near each pole of the neural tube, where one signal is maintained above a certain threshold during the first 25 h, while the levels of the other signal take the lowest values (figure 2). This is consistent with the findings that the duration of signalling influences cell fate decisions. For the middle of the neural tube, where both signals take the lowest values, information also accumulates over time and the probabilities of correct discrimination increase, although to a notably lesser extent resulting in the less marked increase in the peak of the distribution.

**Figure 7.**
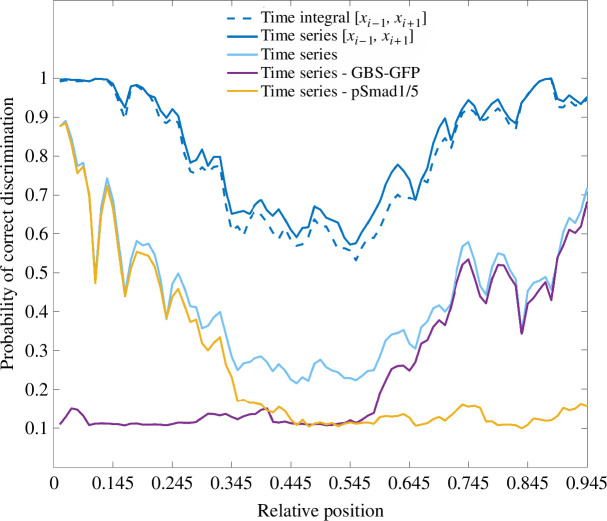
The average probabilities of each of the relative positions being correctly classified in its own bin (where it is not otherwise denoted) or in the region containing the correct bin and its adjacent bins, denoted by $\left[x_{i-1},x_{i+1}\right]$, based on the levels of the signals during the first 35 h, using the time series or the time integral. When the probabilities are obtained using the levels of the two signals separately, the signal used is denoted. Otherwise, they are obtained based on the two signals together.

Observing the time-series probabilities for the 35 h time course obtained from the levels of GBS-GFP and pSmad1/5 activity separately, it is evident that the signalling from the second morphogen contributes significantly to precision along the major part of the neural tube (figure 7). Shh signalling alone provides insufficient information for the region [0.055,0.585] of the neural tube, and BMP signalling provides limited information to [0.445,0.945], where the probabilities of correct discrimination are around 0.1.

### 2.6. Spatial patterns of positional uncertainty

**Figure 8.**
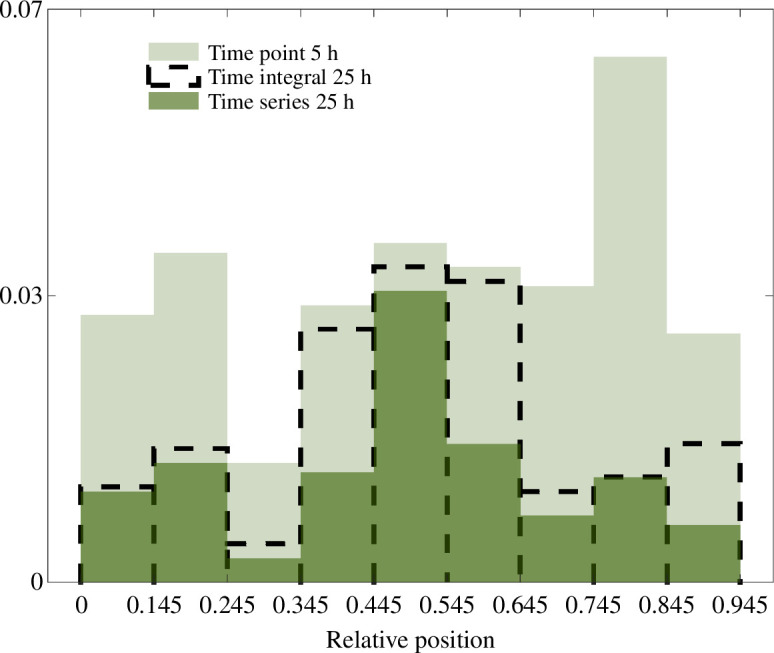
The distributions of incorrectly decoded signal levels obtained at time point 5 h and using the time integral and the time series during the first 25 h. The x-axis shows the relative positions where the incorrectly decoded signal levels are measured and the y-axis the fraction they make out of all the signal levels considered at the given time. The results are obtained on the basis of the two signals together.

A signal level $y_{l}^{i}$ is correctly decoded if the probability distribution $P(X|Y=y_{l}^{i})$ attains its maximum at the correct bin $x_{i}$, and incorrectly if the maximum is at any other $x_{j}$, j≠i. We assume that this maximum is always unique since the system should provide unambiguous positional information. Therefore, the fraction of incorrectly decoded signal levels at time points 5, 15 and 25 h is 32, 30 and 34%, while for the 15, 25, 35 and 65 h time courses it is 16, 12, 11 and 5%, respectively. When we consider the time integrals after the first 15, 25 and 35 h, this fraction is 20, 17 and 19%. Examining the relative positions that are incorrectly decoded at the time point 5 h indicated that these positions are almost uniformly distributed along the axis, with the most incorrectly discriminated positions at the ventral end (figure 8). Taking the time series and the time integral after the first 25 h decreases the number of incorrectly decoded levels in the ventral and dorsal third of the neural tube, while in the middle only the time series results in a significant improvement.

The time-series and time-integral results showed that after 25 and 35 h a correct bin $x_{i}$ is mistaken for a bin adjacent to it, i.e. for $x_{i-1}$ and $x_{i+1}$ (or only $x_{i+1}$ if i=1 and $x_{i-1}$ if i=15). In terms of relative length, this is within the region ±0.06 from the correct bin, which is ∼2.5 cell diameters at 35 h. On the other hand, at the first three time points individually, 10, 15 and 21% of incorrectly decoded signal levels, respectively, are most probable to be in bins that are not adjacent to the correct one. We calculated the average probability of being inside an area including $x_{i-1}$, $x_{i}$ and $x_{i+1}$—the true bin and its nearest neighbours—after observing a signal level measured at each of the considered relative positions, found as the sum $P(X^{*}=x_{i-1}|x^{i,k})+P(X^{*}=x_{i}|x^{i,k})$$+P(X^{*}=x_{i+1}|x^{i,k})$. We denote this sum as $P(X^{*}\in [x_{i-1},x_{i+1}]|x^{i,k})$. Compared to the average probabilities of being in the correct bin, the average probabilities of being in the region $\left[x_{i-1},x_{i+1}\right]$ take considerably higher values. In particular, considering the time integral and the time series over the first 35 h, the average probabilities of being in the region $\left[x_{i-1},x_{i+1}\right]$ are higher than 0.5 for all positions, and for the dorsal region [0.055,0.255] and the ventral region [0.695,0.945] these probabilities are higher than or equal to 0.9 (figure 7). If we assume that $P(X^{*}|X=x^{i,k})$ is a normal distribution with the bin $x_{i}$, whose average absolute length is smaller than 2 cell diameters during the first 30 h, as its mean, we can approximate its standard deviation. At the first four time points individually, the highest errors are found in the middle of the neural tube and are equal to approximately 2, 3, 4 and 7 cell diameters, respectively. At the same time points, in both the time-series and time-integral scenarios, the highest errors are approximately 2, 2, 2 and 3 cells, respectively. This is consistent with the idea that signalling dynamics improves precision and maintains it at approximately the same level during the first 30 h.

## 3. Discussion

The development of the neural tube requires precise spatial patterning along the dorsal–ventral axis. This is governed by Shh and BMP morphogen gradients that provide positional information via intracellular signalling pathways [25]. Using experimentally measured levels [3] of the downstream transcriptional effectors of these signalling pathways—GBS-GFP and pSmad1/5 activity, respectively—we used the SLEMI algorithm [32] to quantify and decode the positional information transmitted by the two pathways. This analysis confirmed that a single morphogen gradient contains insufficient information to accurately resolve positions, except in the most dorsal or ventral regions of the neural tube, adjacent to the source of morphogen. By contrast, the combination of Shh and BMP signalling provides substantially more spatial information. Furthermore, we found that both the temporal trajectories and the time integrals of the signals carried more information than instantaneous snapshots. Although temporal trajectories carry the most information at all times, both methods of encoding information using temporal profiles provide very similar results during the first 35 h. Later only time series contribute new information, although at the cost of high redundant information. However, in both cases, during the first 35 h there is sufficient information for accurate positional estimates, particularly for the dorsal and ventral thirds of the neural tube. Thus, cells would gain significantly more information by tracking signalling over the initial 35 h of neural tube development and this would allow a substantially increased precision of positional decoding in the neural tube. Overall, therefore, tracking the temporal profile of the two signals improved precision, and the analysis is consistent with the temporal profile of the two signals together providing more positional information than either the signals individually or snapshots of the signals.

Nevertheless, in the middle third of the neural tube, where both BMP and Shh signalling is low, the information provided by the measured signalling gradients remained limited. While our analysis focused on Shh and BMP signalling, it is important to note that other morphogens may contribute to neural tube patterning. Retinoic acid signalling from the paraxial mesoderm has been implicated in neural tube patterning, particularly in the intermediate regions and could provide additional positional information [39]. Moreover, the Wnt family of secreted glycoproteins have been suggested to play a role in dorsal neural tube patterning and could provide additional information in conjunction with BMP signalling [40]. The contribution of these additional morphogens to positional information, especially in the middle regions of the neural tube, warrants further investigation and integrating data on these signalling pathways into our information-theoretic framework could provide additional insight into neural tube patterning.

Previously Zagorski *et al*. [3] established a mathematical framework for decoding positional information in the neural tube. This involved several approximations. The probability distribution of signal at a single relative position x, P(Y|X=x), was assumed to be Gaussian. The signals were also assumed to be independent and only individual time points considered. Our approach relaxes these assumptions. Nevertheless, the conclusions are broadly consistent with the previous analysis. Similar to the results reported here, Zagorski *et al*. show that the time-point positional error is highest for the middle of the neural tube and increases over time; they found the highest positional imprecision to be approximately 3, 4, 5 and 8 cells at 5, 15, 25 and 35 h, respectively. Moreover, Zagorski *et al*. conclude that a combination of signalling provides substantially lower positional error throughout the neural tube than either signal individually. The analyses are consistent with previous experimental evidence that the initial 30 h of mouse neural tube development are critical for tissue patterning. Our approach extends the analysis from Zagorski *et al*. by taking series and integrals of signal levels during the initial approximately 30 h to conclude that the signalling over time decreases positional error. Patterning specifies 11 domains of neural progenitors. If these domains were of the same size, the length of each would be approximately 3 cell diameters at 30 h. We found that, at this point in patterning, the accumulated information is sufficient to perfectly distinguish regions containing approximately 6 cells on average. Moreover, the highest error, given by the standard deviation, with which a bin of size less than 2 cell diameters can be specified is approximately 2 cells. Therefore, a domain containing 3 cells could be specified with an error of less than 2 cells. Zagorski *et al*. have measured the boundaries of the two domains of gene expression, Pax6 and Nkx6.1, which are positioned in the middle of the tube. The precision of the boundaries was found to be approximately ±1 and ±2 cells at 30 h. As suggested by Zagorski *et al*. [3], the similarity in precision of signalling profiles and boundary positions indicates that cells decode the information in a way that minimizes imprecision.

Our results show the discrepancy in precision between the middle and the ends of the neural tube, which raises the question if this is potentially compensated for by another source of information or another mechanism for information decoding. The gene regulatory networks downstream of morphogen signalling play a crucial role in the interpretation of positional information [2,25]. It is still unknown how precisely the levels of Shh and BMP signalling are measured downstream. If only thresholds of their concentrations can be sensed [41], the question is how much positional information is used. However, cross-repressive interactions between transcription factors help refine boundaries, as evidenced by shifting, noisy boundaries when specific transcription factors in the downstream network are lost [5]. The design of the network appears to enhance patterning precision by reducing the effects of transient fluctuations in signalling. Extending the information-theoretic approach directly to quantified transcription factor levels could provide further insight into how the gene regulatory network processes time-varying positional information and affects the precision of tissue patterning. In addition, corrective mechanisms such as differential adhesion have been implicated in contributing to patterning precision [24]. Assessing the role such mechanisms play in the precision of neural tube patterning would provide insight into the relative contribution of the information provided by morphogen signalling compared to downstream mechanisms.

The information-theoretic approach provides a powerful framework for analysing morphogen-based patterning that does not rely on detailed mechanistic knowledge of the patterning process. The data-driven nature, however, means that the accuracy of the experimental measurements of signalling dynamics influences the analysis, and the analysis is constrained to the available time and spatial points at which measurements are made. In regions of the tissue where the level of signalling is low, the fluorescent intensity of the corresponding signal reporters is low and approaches background levels; this increases the uncertainty in estimated signalling levels and results in lower estimates of positional information than would be obtained if more sensitive reporters were available. In addition, the temporal trajectories used in our measurements contain variations at the level of the whole population. This means that the computed bits of information do not represent the information provided to an individual single cell, but rather a general profile of positional information available in the system that can be used for patterning. It would be ideal to follow the level of signalling in individual cells in the same embryo over time. This would provide additional insight into how much information cells receive and allow quantification of the noise that arises in a single embryo. Moreover, analysing data from experiments that modulate different aspects of the Shh and BMP signalling pathways could provide a deeper understanding of the information transmission in the system. For example, in Zagorski *et al*. [3], signal gradients are measured in embryos that are hypomorphic for Shh signalling.

How to define position in a growing domain is still an open question [19]. Following a single cell and its movement and division would give us the most accurate picture of the cell’s position over time. The growth in the neural tube has been studied [6,42]. However, how growth is coupled with morphogen signalling and gene regulatory networks remains to be clarified.

Overall, the study supports the conclusion that information theory combined with quantitative dynamic data is a powerful approach to unravel the mechanisms of precise spatial patterning in development, and permits a quantifiable definition of positional information. The temporal integration of morphogen signals is likely crucial for the patterning of many developmental systems, as the duration of morphogen signalling has been implicated in the patterning of other tissues [43–45]. The methodology described in this study could therefore provide insight into other developmental systems. The success will depend on obtaining the appropriate quantitative data and expanding theoretical and experimental knowledge of the systems.

## Data Availability

The data used in this work are from previously published work [3]. They are not publicly available and were provided to us by the authors. Supplementary material is available online at [46].
